# Juvenile fibroadenoma

**DOI:** 10.1590/0100-3984.2016.0162

**Published:** 2018

**Authors:** Décio Roveda Júnior, Gustavo Machado Badan, Mário Sérgio Dantas do Amaral Campos, Bianca Maragno, Laís Bastos Pessanha

**Affiliations:** 1 Santa Casa de São Paulo, São Paulo, SP, Brazil.; 2 Faculdade de Medicina de Campos (FMC), Campos dos Goytacazes, RJ, Brazil

**Figure 1:**
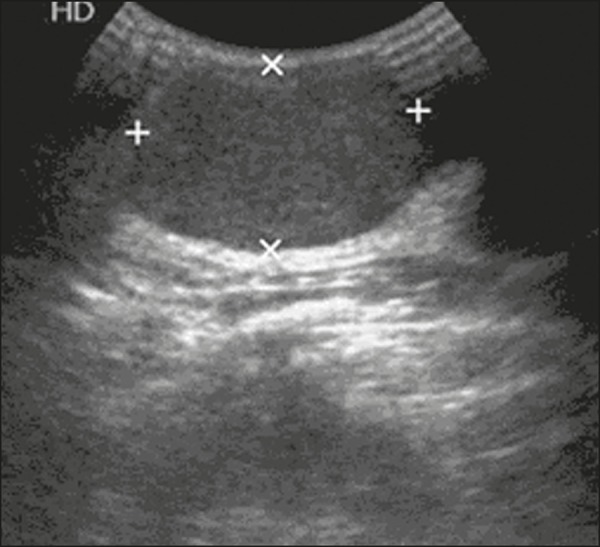
Ultrasound showing oval, circumscribed, hypoechoic nodules, with their longest axis parallel to the skin, suggestive of lesions that are probably benign in nature.

**Figure 2:**
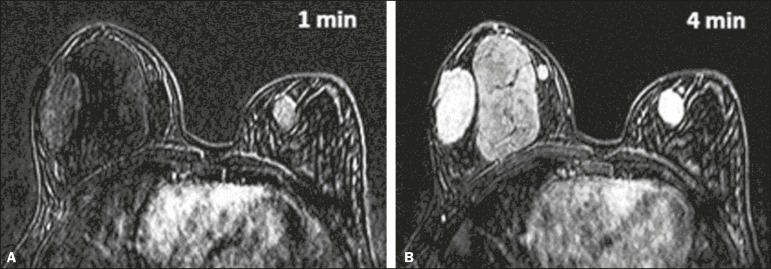
Magnetic resonance imaging identifying lesions with the same morphology described on ultrasound, all of the lesion showing a hypointense or isointense signal in T2-weighted sequences, as well as delayed, progressive enhancement after administration of paramagnetic contrast agent in the T1-weighted sequence, with subtraction from the first minute (A) to the fourth minute (B).

**Figure 3:**
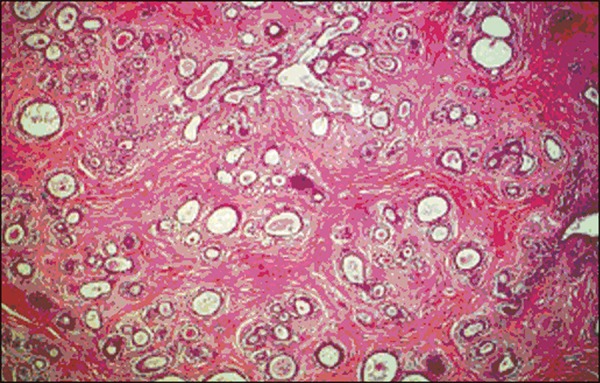
Electron microscopy image showing immature lobules and ducts in clefts, together with proliferation of myoepithelial and stromal cells, findings consistent with juvenile fibroadenoma.

Dear Editor,

A 17-year-old black female presented with palpable nodules in both breasts. Five months prior, she had noticed abrupt growth, consequently undergoing ultrasound (Figure 1) and magnetic resonance imaging (Figure 2). Due to the growth of the lesions over a short period of time, ultrasound-guided core biopsy was requested for a better diagnostic evaluation (Figure 3).

In children and adolescents, most of the clinical conditions that result in an increase in breast size or nodules in the breast are of a benign nature. A unilateral increase in breast size is most commonly related to abnormal breast development, whereas nodules in the breast are most commonly related to fibroadenoma. Such nodules present a low risk of becoming malignant, are hormone-dependent, and can shrink after menopause^(1)^.

Juvenile (or cellular) fibroadenomas, which account for 7-8% of all histological fibroadenoma subtypes, present accelerated growth and have a predilection for young black females^(2,3)^. At diagnosis, 10-25% of juvenile fibroadenoma patients have multiple or bilateral tumors, as in the case presented here. The biological behavior of juvenile fibroadenoma is one of a rapidly growing lesion affecting the breast, some patients showing skin ulceration and superficial venous distention^(3,4)^.

Ultrasound examination is the main tool used in the diagnostic investigation of breast lesions in young patients, being highly sensitive for the detection and monitoring of fibroadenomas. In the vast majority of cases, they have a typical appearance-an oval, circumscribed, hypoechoic nodule, with its longest axis parallel to the skin, with or without vascularization on a Doppler study. In older patients, such nodules can show calcium or necrotic degeneration, mimicking aggressive lesions^(5)^. On magnetic resonance imaging, fibroadenoma can exhibit a variety of behaviors. In the great majority of cases, fibroadenoma lesions show a hypointense or isointense signal in T2-weighted sequences and internal septations; after intravenous administration of paramagnetic contrast medium, the pattern of enhancement can be type I (progressive ascending curve), type II (plateau curve), or absent^(6)^.

The main differential diagnosis of fibroadenoma is a phyllodes tumor, which can be of a malignant or benign nature, making it fundamental to perform biopsy with histological analysis in order to differentiate between the two. Giant fibroadenomas and phyllodes tumor can be indistinguishable by imaging methods^(2-4)^.

Knowledge of the clinical history, the characteristics identified by imaging methods, and the histological correlation with morphologic changes or growth of the nodules of more than 20% over a short period of time provide the tools necessary for radiologists and attending physicians to manage cases of juvenile fibroadenoma appropriately.
